# Supplementary material for: Eligibility and GDMT up-titration success in heart failure: A real-world assessment
Source: PLoS One. 2025 May 27;20(5):e0323952. doi: 10.1371/journal.pone.0323952 (PMC12112052; doi:10.1371/journal.pone.0323952)
Supplement: S1 File — (DOCX) [file pone.0323952.s001.docx]

**Supplementary Figure 1. Use and dosage of HF therapy at baseline, 6 months, and 1 year depending on GDMT up-titration limiting factors. (a) Use and (b) the percent of target dosage** are shown as histograms and Tukey box plots for each profile and time points grouped for the respective substance, i.e., RASi, BB, MRA. The matched Wilcoxon was used for comparisons between baseline vs. 6 months and baseline vs. 1 year. The p-value is indicated as an asterisk in the respective plots.

**
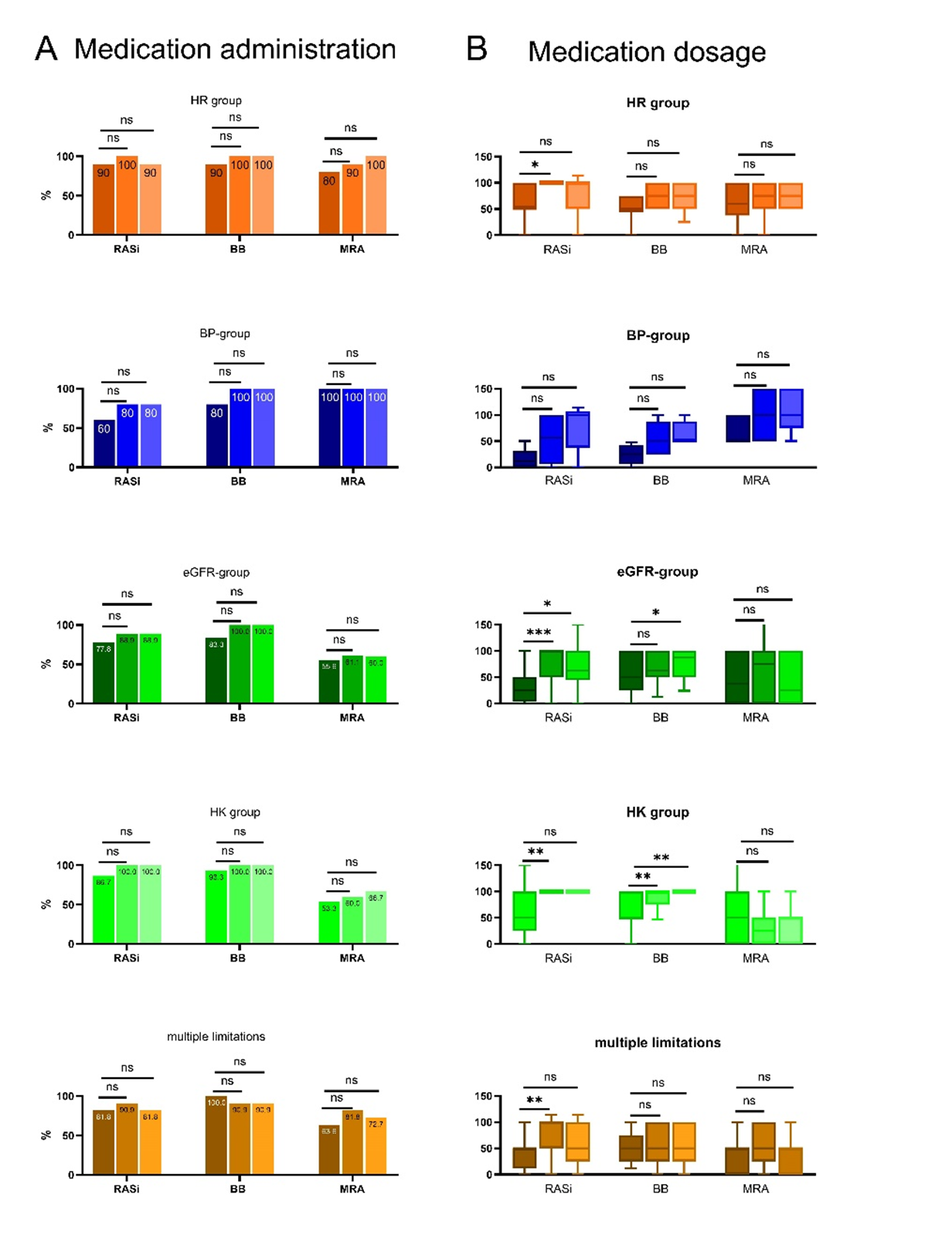
**

*Bar order: Baseline, 6 months, 1 year; NS – p>0.05; * - p≤0.05; ** - p≤0.01; *** - p≤0.001; **** - p≤0.0001; BL - Baseline; BB - Betablocker; RASi - Renin Angiotensin System Inhibition; MRA - Mineralocorticoid Receptor Antagonist*

**Supplementary Figure 2. Non-classifiable patients: characterization, distribution, and survival.**

Characteristics of the non-classifiable HFA profiles of patients according to clinical features are shown in the left panel. 12 large groups were defined as alpha, beta, gamma, and delta with isolated high SBP (n=77), CKD (n=140), significant CKD (n=33), and atrial fibrillation (n=88) as the main traits. Additionally, 142 patients were summarized under “other” as these exhibited a more complex pattern with various combinations of clinical features. A. The distribution of these alternate HFA profiles is shown as a donut chart. B. The association of these HFA profiles with all-cause mortality is shown as Kaplan-Meier plots; the difference between groups was assessed by the log-rank test. The p-value is indicated in the plot.


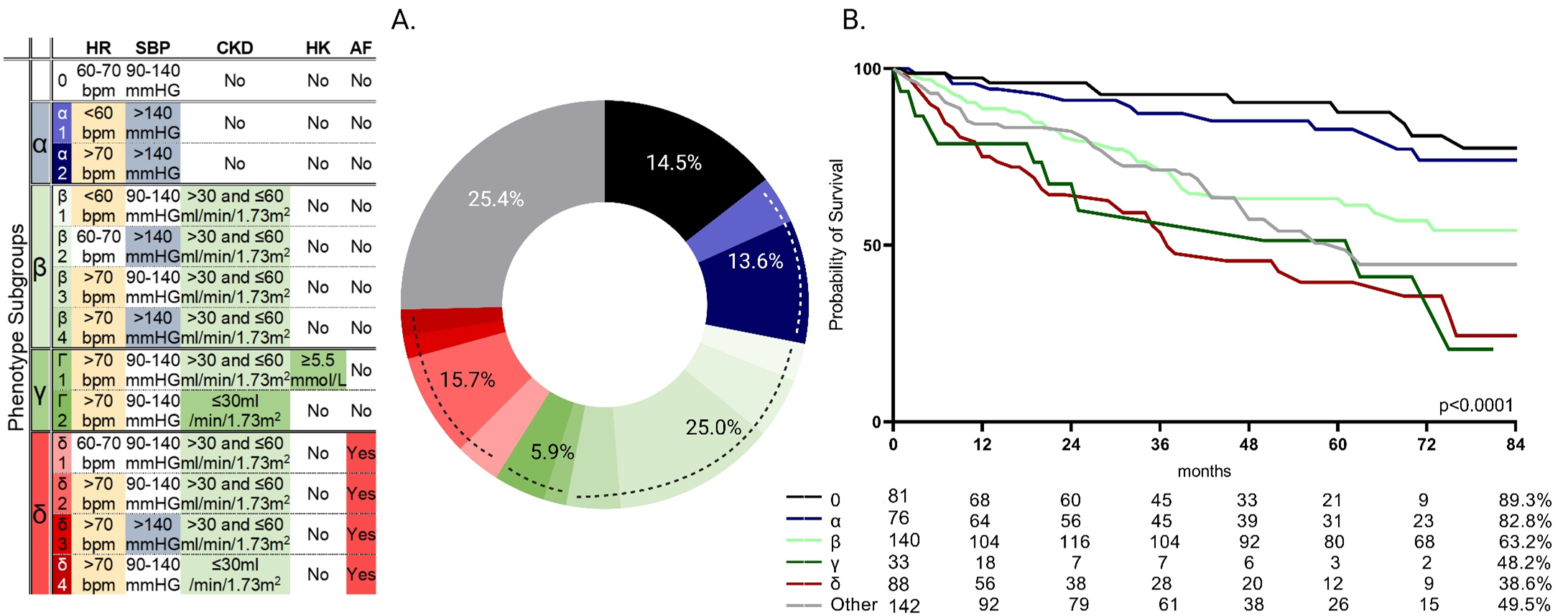


*HR – heart rate; SBP – systolic blood pressure; CKD – chronic kidney disease; HK – Hyperkalemia; AF – atrial fibrillation*

*Other: comprised of multiple combinations of phenotype characteristics where no statement can be made, all with less than 10 patients.*

**Supplementary Figure 3. Use and dosage of HF therapy at baseline, 6 months, and 1 year depending on GDMT up-titration limiting factors. (a) Use and (b) the percentage of target dosage** are shown as histograms and Tukey box plots for each HFA profile and time points grouped for the respective substance, i.e., RASi, BB, MRA. The matched Wilcoxon was used for comparisons between baseline vs. 6 months and baseline vs. 1 year. The p-value is indicated as an asterisk in the respective plots.

*
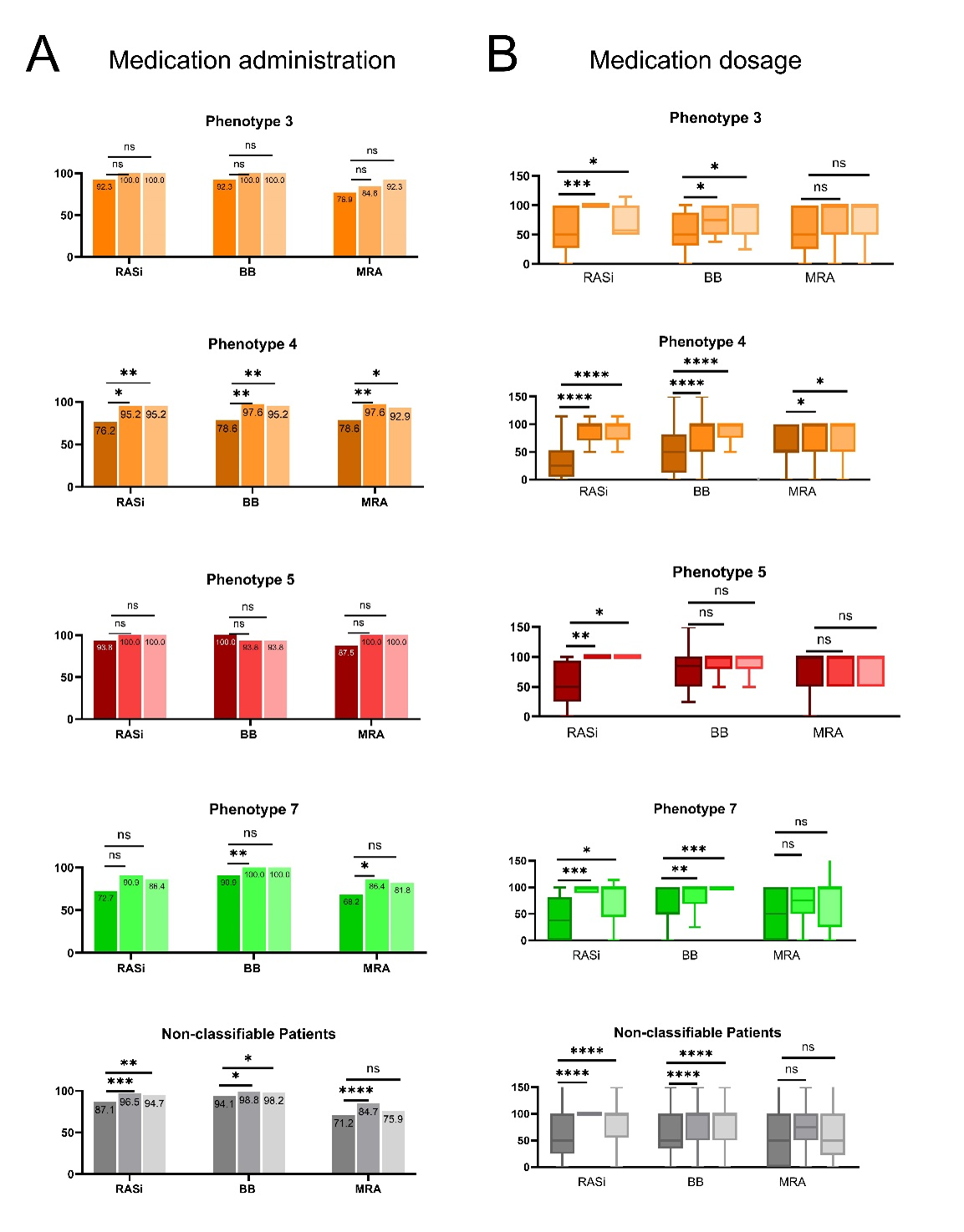
*

*Bar order: Baseline, 6 months, 1 year; NS – p>0.05; * - p≤0.05; ** - p≤0.01; *** - p≤0.001; **** - p≤0.0001; BL - Baseline; BB - Betablocker; RASi - Renin Angiotensin System Inhibition; MRA - Mineralocorticoid Receptor Antagonist; SGLT2i - Sodium Glucose Transporter 2 Inhibition*n

**Supplementary table 1. Modified phenotypes based on the classification by Rosano et al. 5 Adjustments (SBP, 3xCKD, HK) were made.**

| Phenotype 1 | | HR > 70 bpm | SBP < 90 mmHg | No CKD/HK | | No AF | Medication should be adjusted |
| --- | --- | --- | --- | --- | --- | --- | --- |
| Phenotype 2 | | HR < 60 bpm | SBP < 90 mmHg | No CKD/HK | | No AF | Medication should be adjusted |
| Phenotype 3 | | HR < 60 bpm | SBP 90 – 140 mmHg | No CKD/HK | | No AF | Medication should be adjusted |
| Phenotype 4 | | HR > 70 bpm | SBP 90 – 140 mmHg | No CKD/HK | | No AF | Quadruple therapy |
| Phenotype 5 | | HR > 60 bpm | SBP 90 – 140 mmHg | No CKD/HK | | AF | Quadruple therapy |
| Phenotype 6 | | HR 60 – 70 bpm | SBP < 90 mmHg | No CKD/HK | | AF | Medication should be adjusted |
| Phenotype 7 | a | HR 60 – 70 bpm | SBP 90 – 140 mmHg | eGFR ≤ 30 ml/min/1.73 m | No HK | No AF | Medication should be adjusted |
|  | b | HR 60 – 70 bpm | SBP 90 – 140 mmHg | eGFR 30-60 ml/min/1.73 m | No HK | No AF | Quadruple therapy |
|  | c | HR 60 – 70 bpm | SBP 90 – 140 mmHg | eGFR ≤ 60 ml/min/1.73 m | K ≥ 5.5 mmol/L | No AF | Medication should be adjusted |
| Phenotype 8 | | HR 60 – 70 bpm | SBP < 90 mmHg | No CKD/HK | | No AF | Medication should be adjusted |
| Phenotype 9 | | HR 60 – 70 bpm | SBP > 140 mmHg | No CKD/HK | | No AF | Quadruple therapy |
| Non-classifiable | | any other combination | | | | | no suggestions |

HR – heart rate; SBP – systolic blood pressure; CKD – chronic kidney disease (eGFR ≤ 60 mL/min/1.73m^2^); K-Potassium; HK – Hyperkalemia (Potassium ≥ 5.5 mmol/l); AF – atrial fibrillation

**Supplementary Methods 1. The HFA concept: classification of the HF clinical profiles**

The respective clinical features for the different profiles of the HFA position paper(16) were defined according to the original work by resting HR (categories for sinus rhythm (SR): low heart rate: <60bpm; 60–70 bpm and high heart rate: >70 bpm; categories for AF: 60-70 bpm and > 60 bpm), systolic BP (categories: low BP: <90mmHg; normal BP: > 90 mmHg and high BP: > 140 mmHg), presence or absence of chronic kidney disease (CKD) (eGFR < 30 ml/min/1.73m^2^ (categories yes/no) or eGFR < 60 ml/min/1.73m^2^ (categories yes/no)), the presence or absence of hyperkalemia (HK) without an indicated cut-off (categories yes/no) and AF (categories yes/no).(16) Certain adjustments were made to avoid overlapping categories within this definition. i) Patients initially classified with normal blood pressure (systolic BP of >90mmHg) but not meeting the criteria for hypertension (systolic BP of >140 mmHg), were redefined to have a systolic BP of 90-140 mmHg (phenotype 3, 4, 5, 7), to distinguish them from the high BP phenotype (phenotype 9). ii) In the absence of a defined cut-off value for serum Potassium, a threshold of ≥ 5.5mmol/l was established to define HK. iii) Phenotypes characterized by CKD (phenotype 7) were defined as having an eGFR ≤60 mL/min/1.73 m^2^. In case patients had CKD, they were classified into the category with eGFR ≤ 30 mL/min/1.73 m2 and eGFR > 30-≤60 mL/min/1.73 m2 (7a and 7b); in case patients had HK, they were grouped to 7c.

The combination of these traits resulted in the phenotypes described in **Supplementary Table 1**.
