# Supplementary material for: Eligibility and GDMT up-titration success in heart failure: A real-world assessment
Source: PLoS One. 2025 May 27;20(5):e0323952. doi: 10.1371/journal.pone.0323952 (PMC12112052; doi:10.1371/journal.pone.0323952)
Supplement: Attachment — Submitted filename: Plos_One_Review.docx [file pone.0323952.s005.docx]

Dear Dr. Hülsmann,

Thank you for submitting your manuscript to PLOS ONE. After careful consideration, we feel that it has merit but does not fully meet PLOS ONE’s publication criteria as it currently stands. Therefore, we invite you to submit a revised version of the manuscript that addresses the points raised during the review process.


Please submit your revised manuscript by Apr 16 2025 11:59PM. If you will need more time than this to complete your revisions, please reply to this message or contact the journal office at [plosone@plos.org](mailto:plosone@plos.org). When you're ready to submit your revision, log on to https://www.editorialmanager.com/pone/ and select the 'Submissions Needing Revision' folder to locate your manuscript file.

Please include the following items when submitting your revised manuscript:

- A rebuttal letter that responds to each point raised by the academic editor and reviewer(s). You should upload this letter as a separate file labeled 'Response to Reviewers'.
- A marked-up copy of your manuscript that highlights changes made to the original version. You should upload this as a separate file labeled 'Revised Manuscript with Track Changes'.
- An unmarked version of your revised paper without tracked changes. You should upload this as a separate file labeled 'Manuscript'.

If you would like to make changes to your financial disclosure, please include your updated statement in your cover letter. Guidelines for resubmitting your figure files are available below the reviewer comments at the end of this letter.

If applicable, we recommend that you deposit your laboratory protocols in protocols.io to enhance the reproducibility of your results. Protocols.io assigns your protocol its own identifier (DOI) so that it can be cited independently in the future. For instructions see: <https://journals.plos.org/plosone/s/submission-guidelines#loc-laboratory-protocols>. Additionally, PLOS ONE offers an option for publishing peer-reviewed Lab Protocol articles, which describe protocols hosted on protocols.io. Read more information on sharing protocols at <https://plos.org/protocols?utm_medium=editorial-email&utm_source=authorletters&utm_campaign=protocols>.

We look forward to receiving your revised manuscript.

Kind regards,

Satoshi Higuchi
Academic Editor
PLOS ONE

We thank the Editorial Board for the approval of our work and the opportunity to resubmit a revised version of our manuscript to PLOS ONE. Furthermore, we would like to thank the reviewers for the constructive comments, which have stimulated us to make this, as we think, a much better paper. Please find a detailed response to the reviewer’s comments below.

Journal Requirements:

When submitting your revision, we need you to address these additional requirements.

REVIEWER COMMENTS:
================

Reviewer 1:

The study investigates the presence of limiting factors in the gradual titration of GDMT in patients with advanced HFrEF in real clinical settings and its impact on the establishment of optimal pharmacotherapy. It presents very interesting findings.

However, I feel that several revisions are needed for the manuscript, as detailed below.

We thank the reviewer for the thoughtful review of our manuscript and for recognizing the general significance of our work and its contribution to the field. We are committed to addressing the issues that were pointed out and are sure that it will enhance the significance of our manuscript.

1. The cohort in this study is derived from a single tertiary medical center. How much do the results differ from those in a more general population of HFrEF patients? To what extent are the findings generalizable?

The cohort in this study is derived from a single center tertiary clinic, which may limit the generalizability of the findings. Patients at tertiary centers tend to have more severe disease and receive more specialized care compared to the general population of HFrEF patients.

We have compared our results with data from larger, multi-center registries, i.e. the CHAMP-HF registry^1^ and Swede-HF registry^2^. The CHAMP-HF registry includes outpatients in the US with chronic HFrEF. The SwedeHF registry^2^ is a comprehensive national quality register in Sweden, including patients with chronic heart failure. The key characteristics of the three registries are displayed in Revision Table 1.

Revision Table 1. HFrEF patient characteristics across national registries.

| **Characteristics** | CHAMP-HF | SWEDE-HF | Our Cohort |
| --- | --- | --- | --- |
| Mean/Median Age, years | 66±13 | 75 | 64 (Q1-Q3: 53-73) |
| Gender, male % | 71% | 65% | 76.3% |
| NYHA class III-IV, % | 32% | 40% | 39.6% |
| Median NT-proBNP, pg/ml | ~1840 pg/ml- | ~2630 pg/ml | 1951 (Q1-Q3: 814 – 4328) |
| Coronary artery disease, % | 40% | 50% | 44% |
| Atrial fibrillation, % | High prevalence | 40% | 31% |
| Diabetes Mellitus, % | 40% | 30% | 33% |
| **Medication use at baseline** |  |  |  |
| RASi, % | 13% | 91% | 85% |
| MRA, % | 25% | 42% | 68% |
| Beta-Blocker, % | 20 | 92 | 89% |
| Up-titration assessed after, months | 12 months | Not uniformly defined | 6 months, 12 months |
| Triple Therapy at target dosages at FUP, % | 1% | Not calculated for triple therapy | 35% |

The Vienna HFrEF cohort seems to be comparable with the population studied in CHAMP-HF and SWEDE-HF – the cohort has similar age as CHAMP-HF, NYHA class, NTproBNP levels and comorbidities are comparable to SWEDE-HF. Technically, physician had no additional access to other diagnostics or therapeutics compared to other office care. The essential difference is the intensive management and close monitoring provided in our center may not be feasible in all settings. Our center probably buys more intensive treatment through more frequent visits. This point is now also added in the paragraph limitations.

We will incorporate this into the manuscript, as suggested.

*Discussion:*

This finding is of particular significance as the data were obtained from a tertiary care center, where patients are typically referred in case they are at an advanced state of HF or if the treating physician deems therapy initiation or up-titration unfeasible due to other reasons. However, the comparability of our cohort with the populations studied in the CHAMP-HF and SWEDE-HF registries suggests that our findings are generalizable to the broader HFrEF population, despite being derived from a tertiary care center.

2. There is no clear mention of the limitations of the study.

We acknowledge the limitations in our study and will mention them in the text:

*Limitations:*

Limitations: Our study was conducted retrospectively at a single tertiary medical care center, which may limit the generalizability of our findings. Although we achieved a high proportion of patients reaching target dosages for triple therapy, this may not be reflective of broader clinical practice. The intensive management and close monitoring provided in our center may not be feasible in all settings, potentially limiting the reproducibility of our results.

3. In the Supplementary Methods (Section 1), it is stated that for AF, resting HF is categorized as 60-70 and >60. Is this correct?

Yes, this is correct, and corresponds to the HFA profiles (Revision Figure 1).

Revision Figure 1. HFA profiles according to Rosano et al.(PMID: 33932268)

These are the only two profiles for AF patients. We are aware that the distinction for AF patients solely based on these HR categories, i.e. 60-70bpm and >60bpm, generates an overlap, yet these two profiles also differ in terms of BP, i.e. BP >90/60mmHg for HR >60bpm and BP <90/60mmHg for HR 60-70bpm. So that there is no overlap between the two profiles but rather AF patients may not be reflected by these profiles, e.g. AF patients with BP <90/60mmHg and HR >70bpm or a HR <60bpm. This also applies for AF patients with CKD or hyperkalemia, these patients cannot be categorized into any HFA profile.

The fact that patients actually may not be categorized into any HFA profile is one of the main confirmatory findings of our paper. As the HFA profiles use 5 clinical factors divided into 2-3 categories, all possible variations of these categories would result in 108 profiles.^3^ Only around 40% of patients are reflected by the 11 profiles proposed in Rosano et al. paper, which should be the most commons according to the authors. However thereby partly more healthy but also the sickest patients are missed. Treatment recommendations would be most crucial for the latter group.

4. Was the actual adjustment of medications based on the cut-off values for the GDMT titration limiting factors as specified in this study, or was it left to the discretion of the attending physicians? For instance, is it possible that RASI medications were up-titrated even when the eGFR was less than 30?

The reviewer is right. The adjustment of medication was left to the discretion of the attending physician. The data in this manuscript are derived from our prospective registry, most of that data was generated before the introduction of the HFA profiles. We wanted to show how real life data on GDMT implementation is congruent with the proposed HFA profiles. We wanted to know how applicable the HFA profiles were and whether distinct profiles would end up with different medication without previously classifying them into those HFA profiles. The caution of titrating RASi in patients with an eGFR <30ml/min/1.73m^2^ is derived from the RCTs where patients with a lower eGFR were excluded. So, there is simply no evidence in this eGFR range. Retrospective and observational data however suggest a preserved benefit for RASi and particularly MRA for lower eGFR ranges.^4,5^ So that during the forced GDMT implementation attempt RASi and MRA would be up-titrated in cases that eGFR is slightly lower than 30ml/min/1.73m^2^.

5. In the first paragraph of page 12, it is written: "v) the HFA profiles need to be adapted, as all patients defined by the HFA profiles can be successfully up-titrated while the most severe patients are missed by the classification and, thereby require specific treatment recommendations." In the position paper, however, it states: "Of course, physicians will recognize patients cannot always be characterized accurately by simple demographics, so that advice may need to be sought by comparison and combinations of the advice for one or more profiles." The statement in the manuscript feels somewhat assertive. Can you propose a new classification to identify high-risk patients who are not included in the HFA profiles?

Our data indicate that 62% of patients could not be classified into existing HFA profile, including many of the most severe cases. This suggests that the current HFA classification may not adequately capture high-risk patients. We do not see a clear benefit in further classification, as increasing complexity in profiles does not necessarily lead to better treatment decisions. The ultimate goal should be the best implementation of GDMT, whenever possible. We believe that generating a complex classification would rather discourage physicians from an up-titration attempt. A low sBP or renal impairment should not automatically exclude patients from GDMT. A more individualized yet pragmatic approach appears to be the most effective way to maximize GDMT application.

Reviewer 2:

Overall, the authors present work detailing real world experiences with GDMT use in patients with HFrEF. The author should be commended for completing this important work, as this problem remains central to continued poor outcomes among those with HFrEF.

We thank the reviewer for the detailed feedback and appreciate the recognition of our work. We carefully address each of the points raised below.

There are grammatical errors throughout the text that should be fixed. Additionally, there are a few language syntax errors that could be improved to better portray the message of the authors.

While we acknowledge minor grammatical and syntactical improvements could enhance readability, the scientific content remains intact. We will conduct a careful language review to refine expression.

-------
Introduction – Please abbreviate GDMT in the first instance.

Additionally, it would be valuable to mention HFrEF in the first sentence to set the stage for the rest of the paper, especially given the lack of substantial class I indicated medications for other types of HF.

We will ensure that GDMT is abbreviated upon first mention and will explicitly introduce HFrEF in the opening sentence to provide immediate context.

*Introduction*

Guidelines for the management of patients with Heart Failure with reduced ejection fraction (HFrEF) emphasize the use of all class I recommended drugs, i.e., Guideline directed Medical Therapy (GDMT), with early treatment initiation and up-titration to maximum dosages.

In the second paragraph, the sentence starting with “Other Data” should be referenced so the reader can refer to the data highlighted by the authors.

Thank you for your valuable feedback. We have now referenced the 'Other Data' section accordingly to ensure clarity for the reader.

*Introduction:*

Other data on consecutive hospitalized HF patients show that 71.6% are eligible for up-titration of all GDMT classes.[11]

Additionally, the second sentence on page 5 “This study has used different” is confusing and unclear what point is being made. Was it the Greene study that utilized different cut-offs? Did the study just demonstrate the different cutoffs used in standard practice? Does GWTG data record why people did not receive medications?

Thank you for your comment. The different cut-offs mentioned in this sentence refer to the thresholds applied in the large RCTs that tested and led to the introduction of specific HF medications into clinical practice. These cut-offs often vary.

Revision Table 2. Different cut-offs for side effects / clinical factors limiting GDMT uptitration in large HF RCTs.

| **Trial Name** | **Medication Class** | **Medication** | **Titration Cut-offs** | **Blood Pressure Cut-off** | **Heart Rate Cut-off** | **eGFR Cut-off** | **Potassium Cut-off** |
| --- | --- | --- | --- | --- | --- | --- | --- |
| CONSENSUS^6^ | RASi | Enalapril | Titrated to 20 mg 1-0-1 | > 90 mmHg | - | > 30 mL/min/1.73m² | < 5.5 mmol/L |
| SOLVD^7^ | RASi | Enalapril | Titrated to 10 mg 1-0-1 | > 90 mmHg | - | > 30 mL/min/1.73m² | < 5.5 mmol/L |
| CHARM^8^ | RASi | Candesartan | Titrated to 32 mg 1-0-0- | > 90 mmHg | - | > 30 mL/min/1.73m² | < 5.5 mmol/L |
| Val-HeFT^9^ | RASi | Valsartan | Titrated to 160 mg 1-0-0 | > 90 mmHg | - | > 30 mL/min/1.73m² | < 5.5 mmol/L |
| RALES^10^ | MRA | Spironolactone | Titrated to 50 mg 1-0-0, | > 90 mmHg | - | > 30 mL/min/1.73m² | < 5.0 mmol/L |
| EMPHASIS-HF^11^ | MRA | Eplerenone | Titrated to 50 mg 1-0-0 | > 90 mmHg | - | > 30 mL/min/1.73m² | < 5.0 mmol/L |
| CIBIS-II^12^ | BB | Bisoprolol | Titrated to 10 mg 1-0-0 | > 90 mmHg | HR > 60 bpm | > 30 mL/min/1.73m² | < 5.5 mmol/L |
| COMET^13^ | BB | Carvedilol | Titrated to 25 mg 1-0-1 | > 90 mmHg | HR > 60 bpm | > 30 mL/min/1.73m² | < 5.5 mmol/L |
| MERIT-HF^14^ | BB | Metoprolol succinate | Titrated to 200 mg 1-0-0 | > 90 mmHg | HR > 60 bpm | > 30 mL/min/1.73m² | < 5.5 mmol/L |
| PARADIGM-HF^15^ | ARNI | Sacubitril/Valsartan | Titrated to 97/103 mg 1-0-1 | > 100 mmHg | - | > 30 mL/min/1.73m² | < 5.4 mmol/L |
| DAPA-HF^16^ | SGLT2i | Dapagliflozin | Titrated to 10 mg 1-0-0 | > 95 mmHg | - | > 30 mL/min/1.73m² | < 5.5 mmol/L |
| EMPEROR-Reduced^17^ | SGLT2i | Empagliflozin | Titrated to 10 mg 1-0-0 | > 95 mmHg | - | > 20 mL/min/1.73m² | < 5.5 mmol/L |
| STRONG-HF^18^ | Various | Various | Intensive titration to 100% of recommended doses within 2 wees post-discharge | > 90 mmHg | - | > 30 mL/min/1.73m² | < 5.5 mmol/L |
| SHIFT^19^ | Ivabradine | Ivabradine | Titrated to 7.5 mg 1-0-1 | > 85 mmHg | HR >70 bpm | - | - |
| ATLAS^20^ | RASi | Lisinopril | Titrated to 35 mg 1-0-0 | >90 mmHg | - | >20 mL/min/1.73m² | - |
| TOPCAT^21^ | MRA | Spironolactone | Titrated to45 mg 1-0-0 | >90 mmHg | - | >30 mL/min/1.73m² | <5.0mmol/L |

*Introduction:*

This study has used different cut-offs for bradycardia (resting heart rate (RHR) ≤50bpm), systolic blood pressure (≥85-100mmHg), renal function (eGFR ≤30ml/min/1.73m2) and potassium levels (≤ 5.0 or 5.4 mmol/l) as a demonstration for the arbitrariness of what clinicians consider as a contraindication. The different cut-offs mentioned here refer to the thresholds applied in the large RCTs that tested and led to the introduction of specific HF medications into clinical practice, which often vary.

Was the aim of the paper just to look at up-titration of GDMT or was it to evaluate the HFA phenotypes impact or association with up-titration?

The aim of the paper is to investigate the applicability of the proposed HFA profiles on real life data for GDMT implementation. The data in this paper come from a registry, whereas most data is older than the introduction of the HFA profiles. We wanted to know whether the distinct profiles would end up with different medication, while physicians previously did not classified patients into the HFA profiles. The clinic uses a pragmatic approach in implementing GDMT. The study shows that even that patients have different HFA profiles they can be successfully up-titrated and receive comparable GDMT after 1 year, so that probably classification is of limited value in these patients. The study also reveals, that patients with highest risk, who are probably most complex to titrate, are not reflected by the HFA profiles.

Methods – What does an “all-comer” clinic mean? Who gets referred there? People after a hospitalization or people from the community? Is the clinic staffed by cardiologists or heart failure trained cardiologists? APPs?

The term "all-comer" refers to an open-access, specialized HF outpatient clinic that accepts referrals from both hospital-based and community-based cardiologists, as well as internists managing complex HF patients. No specific referral criteria are needed except of recently documented servery reduced LVEF and severely elevated NTproBNP. This clinic is staffed by HF-trained cardiologists and serves as a tertiary referral center for patients requiring comprehensive HF management, with a particular focus on advanced therapy evaluation. The clinic provides assessments, individualized GDMT optimization, and if needed, evaluation for advanced therapeutic strategies, including mechanical circulatory support, heart transplantation, or palliative care in cases of refractory HF.

Does the definition offered in the patient description actually represent “advanced” HF? A low ejection fraction and elevated BNP just means they have HFrEF, not necessarily “advanced” HF, which is usually represented by persistent symptoms despite adequate therapy.

It's important to note that there is no uniform definition of heart failure (HF). Different organizations have their own criteria for defining advanced HF, some criteria are not really objective. Below are the definitions from the European Society of Cardiology (ESC) and the American Heart Association (AHA)/American College of Cardiology (ACC).

In our study cohort, we have patients who meet the criteria for advanced HF according to both definitions, as well as some who do not. However, the majority of our patients do fall within the advanced HF criteria. Therefore, we have defined our cohort based on these widely accepted definitions.

| **Criteria** | **ESC Definition** | **AHA/ACC Definition** |
| --- | --- | --- |
| Symptoms | Persistent symptoms despite GDMT | Severe symptoms and/or signs of HF at rest |
| Hospitalizations | Recurrent hospitalizations for HF | Recurrent hospitalizations despite GDMT |
| Response to Therapy | Intolerance to standard HF medications | Refractory or intolerant response to GDMT |
| Advanced Interventions | Consideration for advanced therapies such as transplant or mechanical support | Consideration for advanced therapies such as transplant, mechanical circulatory support, or palliative care |
| Biomarkers | Elevated NT-proBNP levels | Elevated NT-proBNP levels |

The patients in this paper have persistent symptoms and significantly elevated NTproBNP as an indicator of severe disease. However, we agree with the reviewer, that they may not fall into the generally acceptable definition of “advanced HF”, so that we have changed the wording.

*Abstract:*
This study aims to assess the eligibility for GDMT up-titration and its success in the context of clinical profiles in chronic severe HFrEF outpatients.

In this real-world study 75% of patients with chronic severe HFrEF are eligible for GDMT up-titration towards target dosages

*Introduction:*
The aim of this study was to investigate the proportion of patients eligible for GDMT up-titration and up-titration success in outpatients with chronic severe HFrEF after referral to a specialized HF center.

*Methods:*
The tertiary care outpatient HFrEF clinic of the Medical University of Vienna is an all-comer unit for patients with a recent history of chronic severe systolic HF

*Discussion*:
To our knowledge, this is the first study to investigate the presence of putative GDMT up-titration limiting factors and their impact on establishing optimal medical therapy in a real-world setting in chronic severe HFrEF patients.

**Chronic severe HFrEF and GDMT Eligibility.** In line with these data, also in chronic severe HFrEF 75% of patients in our dataset were evidently eligible for GDMT up-titration at baseline

Conclusion:
This real-world study underlines the feasibility of up-titration of HF treatment in significantly more patients than might be presumed based on registries. Confirming other data, even in chronic severe heart failure 75% of real-world HFrEF patients are eligible for GDMT up-titration at the initial presentation and throughout the up-titration process.xx

In the introduction, the authors introduce the strong-HF exclusion criteria as a source of confusion regarding uptitration success but then cite the same paper in their methodology for developing the “limiting factors” component. Maybe in the introduction, they could reword this reference to something along the lines of, “Implementation trials, like STRONG-HF, use established cutoffs for up titration of medication, and these present challenges to providing adequate therapy.” This could help introduce the commonly help “cut-offs” that will ultimately be used for this analysis.

Thank you for this suggestion. We agree that the wording in the introduction could be refined to better clarify our point. Our intention was not to criticize the use of STRONG-HF exclusion criteria, but rather to highlight that predefined cut-off, which, while it is necessary for trial settings, may pose challenges in real-world implementation. We will revise the introduction to reflect this more clearly, following your suggestion.

*Introduction:*

However, reasons for refraining from up-titration are not precisely defined and may be subject to personal bias. Implementation trials like STRONG-HF, use cutoffs for up-titration of medication, and these present challenges to providing adequate therapy. STRONG-HF has proven that rapidly sequenced up-titration of HF drugs is feasible and safe in patients hospitalized for acute HF. However, here, patients with an estimated glomerular filtration rate (eGFR) < 30 ml/min/1.73m^2^, systolic blood pressure (BP) < 100 mmHg, heart rate < 60 bpm, serum potassium > 5.0 mEq/L and a documented intolerance to high doses of beta-blockers (BB) and Renin-Angiotensin System Inhibitors (RASi) (Angiotensin-converting enzyme inhibitors (ACEi) and Angiotensin receptor blockers (ARB)) were excluded.[10] These predefined cutoffs are essential in trial settings but may pose challenges in real-world implementation, where more flexibility is often required to optimize therapy.

How did the authors determine target doses? Is this from the trial data for each of the medication classes?

For each medication, we referred to GDMT target doses as established in major clinical trials that demonstrated their efficacy. (Revision Table 3). Target dosages were indicated in % for each substance to make the quality of up-titration comparable (e.g. Bisoprolol has a TD of 10mg per day so that 5mg 1-0-1 will be 100% of TD while 5mg 1-0-0 will be 50% etc.)

Revision Table 2. Evidence based doses of disease modifying drugs: ^22^

Analysis – Were KM curves done for each individual limiting factor, or whether a participant had ANY limiting factor vs. NONE. “GDMT at 6 months was calculated in comparison to all patients with data at 6 months” is unclear and the reader may have a hard time understanding what comparison was done here.

The phrase “GDMT at 6 months was calculated in comparison to all patients with data at 6 months” means that up-titration success was assessed only in those with follow-up data. This ensures an accurate comparison without introducing selection bias. We will refine the wording for both issues.

*Methods:*

The survival of patients with and without up-titration limiting factors at baseline was visualized using Kaplan-Meier curves, and a comparison was performed using the log-rank test between all groups

The Boxplot: GDMT at 6 months was calculated ~~in comparison to all patients~~ ~~with~~ from all patients with data at 6 months.

Results – the phrase “clearly eligible” should be defined. Does this mean having no limiting factors?

“Clearly eligible” refers to patients without any limiting factors impeding GDMT up-titration. We will explicitly define this in the Results section.

*Results:*

At baseline, 677 of 900 patients (75.2%) were clearly eligible for implementation and up-titration of GDMT by showing no factor limiting up-titration, while 24.8% showed at least one up-titration limiting factor as defined by convention.

The HFA profiles/phenotypes/etc language throughout the manuscript is confusing. It would be helpful to refer to these profiles with one common name as to not confuse the reader.

Thank you for your feedback. We intentionally used both terms to differentiate between the original HFA classification by Rosano et al., i.e. "profiles," and our own categorization in the second part of the analysis. This distinction was made to avoid confusion and ensure clarity when comparing our findings to the existing framework. However, we acknowledge the concern and will consider streamlining the terminology where possible to enhance readability.

In the intro, it is introduced as a 9 profile system, but in the results, there are 108 different profiles? It would be helpful to spend the time explaining this concept in the methods given that it is a major part of the analysis plan introduced by the authors.

Thank you for your suggestion. We will clarify this in the corresponding section.

The HFA classification system, as suggested in the consensus document, consists of 9, as mentioned in the text, or 11, as shown in the manuscript, profiles. These profiles result from the combination of different clinical factors. Specifically, 5 clinical factors are incorporated into the classification system: the presence of atrial fibrillation (AF) and hyperkalaemia (HK) (each with 2 categories: yes/no), and the values of eGFR, systolic blood pressure (BP) and resting heart rate (RHR) (each with 3 categories: eGFR: <30ml/min/1.73m^2^, 30-60ml/min/1.73m^2^ and no CKD; BP: <90mmHg, 90-140mmHg, >140mmHg; RHR: <60bpm, 60-70bpm, >70bpm). All possible variations of these groups of different clinical features result in a total of 108 profiles.

The HFA chose these profiles, whereas some profiles summarize several profiles, based on their clinical relevance and frequency of occurrence in clinical practice.^23^ However, there was no evidence to support whether these profiles were indeed the most relevant or frequent. Therefore, we aimed to investigate this aspect as well. Our findings showed that more than 60% of patients could not be classified into these profiles, indicating that the chosen profiles may not be as practical or applicable as initially thought. A pervious study has examined the 108 mathematically possible combinations of clinical factors in HF, highlighting the importance of considering all possible variations to optimize patient care.^3^

Therefore, it was important to analyse real-world data to assess the practicality and applicability of these profiles. This was our first research question. Additionally, our study aimed to determine whether these profiles show different outcomes after 1 year of GDMT up-titration without classification. If there is a difference, it would be valuable to know these profiles beforehand. If not, then it may not be necessary

*Results:*

*Figure 4* depicts the distribution of the HFA profiles of the study cohort (The mathematical number of all combinations of HFA clinical feature categories results in 108 possible profiles).

Additionally, how useful is the criteria when >60% of participants are not defined?

We fully agree and this is one of our most remarkable results. Our findings show that >60% of patients do not fit into a predefined HFA profile, which raises concerns about the practical utility of this classification in a real-world setting. Given that phenotyping is intended to help treatment decisions, a system excluding the majority of patients may have limited clinical applicability. Rather than refining the classification, our results suggest that a rigid phenotyping approach may not be necessary, as GDMT up-titration can be successfully implemented without it.

Discussion – It would be helpful for the authors to better clarify why the population studied was “advanced” HF. The criteria presented earlier was not a strong representation of advanced disease, but if there is other evidence that the definition proposed by the authors is reasonable, then it would be helpful to cite.

As noted earlier, our cohort represents a high-risk population typically seen in tertiary HF centres, where patients are referred for advanced therapy evaluation due to persistent symptoms, frequent hospitalizations, and intolerance to GDMT. These characteristics align with established definitions of advanced HF, as outlined by major guidelines, and justify our classification without requiring further refinement. But, we agree, that there is no clear definition and therefore we avoided the term advanced as it is not essential for the results.

On page 13 of the discussion, the authors reference the “treating physician’s dedication and patients’ compliance,” as the contributing factors to lack of titration given the broad eligibility of the patients studied. This point could be strengthened if the authors add greater detail to the patient mix. Are these patients post-hospitalization? Are they from ambulatory clinics? Are these new HF patients or do they have prevalent disease?

Our statement about “physician dedication and patient compliance” is not speculative but a direct interpretation of our data, which show that a significant proportion of patients were eligible for GDMT but did not reach target doses. This highlights real-world barriers to optimal HF therapy, which extend beyond clinical eligibility alone. To provide further context, our cohort includes both post-hospitalization patients and ambulatory referrals, reflecting a heterogeneous mix of newly diagnosed and prevalent HF cases. Patients were referred to our tertiary HF center for specialized evaluation, often due to persistent symptoms, frequent decompensations, or challenges in GDMT optimization at the primary or secondary care level. These characteristics align with what is observed in advanced HF populations, where medication up-titration is frequently limited by factors such as hypotension, renal dysfunction, chronic kidney disease, and intolerance to higher GDMT doses.

What does the final sentence mean in the conclusion? Do the other 25% of patients have alternating limiting factors between time points? How are they not tied to the patient journey? Does journey mean worsening outcomes?

The final sentence refers to the dynamic nature of GDMT-limiting factors. Patients do not remain in a fixed category but transition over time. This transition includes both the resolution of up-titration limiting factors and the development of new ones. This finding is particularly important as it shows that a single point assessment and documentation of an up-titration limiting factor does not necessarily mean that the patient should be withheld from GDMT. Reassessments and rechallenges are crucial.

Whether the resolution or development of up-titration limiting factors is associated with different outcomes is not intuitively clear. On one hand, the resolution of up-titration limiting factors could be due to an improvement in HF in general (e.g., higher blood pressures) but also due to the down-titration of HF therapy. On the other hand, the development of up-titration limiting factors could be due to worsening HF or the initiation or up-titration of HF therapy (e.g., slight decrease in eGFR or blood pressure). Both scenarios have divergent impacts on outcomes. For ARNi and SGLT2i, it is known that, for example, a decline in eGFR, when within a range of 30%, is still associated with better outcomes.^24^ Further studies could evaluate this interesting question. We will clarify this in the text.

*Conclusion:*

In 25% of patients, there is a considerable transition between GDMT up-titration limiting factors, which do not seem to be tied to an individual patient´s journey, but appear dynamic and evolving, highlighting the need for continuous reassessment

References in the Review:

1	Greene, S. J. *et al.* Medical Therapy for Heart Failure With Reduced Ejection Fraction. *JACC* **72**, 351-366 (2018). <https://doi.org:doi:10.1016/j.jacc.2018.04.070>

2	Shahim, A. *et al.* Implementation of guideline-recommended therapies in heart failure with reduced ejection fraction according to heart failure duration: An analysis of 55 581 patients from the Swedish Heart Failure (SwedeHF) Registry. *Eur J Heart Fail* (2025). <https://doi.org:10.1002/ejhf.3565>

3	Musella, F. *et al.* Patient profiles in heart failure with reduced ejection fraction: Prevalence, characteristics, treatments and outcomes in a real-world heart failure population. *Eur J Heart Fail* **25**, 1246-1253 (2023). <https://doi.org:10.1002/ejhf.2892>

4	Matsumoto, S. *et al.* Mineralocorticoid Receptor Antagonists in Patients With Heart Failure and Impaired Renal Function. *J Am Coll Cardiol* **83**, 2426-2436 (2024). <https://doi.org:10.1016/j.jacc.2024.03.426>

5	Bhandari, S. *et al.* Renin-Angiotensin System Inhibition in Advanced Chronic Kidney Disease. *N Engl J Med* **387**, 2021-2032 (2022). <https://doi.org:10.1056/NEJMoa2210639>

6	Effects of enalapril on mortality in severe congestive heart failure. Results of the Cooperative North Scandinavian Enalapril Survival Study (CONSENSUS). *N Engl J Med* **316**, 1429-1435 (1987). <https://doi.org:10.1056/nejm198706043162301>

7	Yusuf, S., Pitt, B., Davis, C. E., Hood, W. B. & Cohn, J. N. Effect of enalapril on survival in patients with reduced left ventricular ejection fractions and congestive heart failure. *N Engl J Med* **325**, 293-302 (1991). <https://doi.org:10.1056/nejm199108013250501>

8	Young, J. B. *et al.* Mortality and morbidity reduction with Candesartan in patients with chronic heart failure and left ventricular systolic dysfunction: results of the CHARM low-left ventricular ejection fraction trials. *Circulation* **110**, 2618-2626 (2004). <https://doi.org:10.1161/01.Cir.0000146819.43235.A9>

9	Cohn, J. N. & Tognoni, G. A randomized trial of the angiotensin-receptor blocker valsartan in chronic heart failure. *N Engl J Med* **345**, 1667-1675 (2001). <https://doi.org:10.1056/NEJMoa010713>

10	Pitt, B. *et al.* The effect of spironolactone on morbidity and mortality in patients with severe heart failure. Randomized Aldactone Evaluation Study Investigators. *N Engl J Med* **341**, 709-717 (1999). <https://doi.org:10.1056/nejm199909023411001>

11	Zannad, F. *et al.* Eplerenone in patients with systolic heart failure and mild symptoms. *N Engl J Med* **364**, 11-21 (2011). <https://doi.org:10.1056/NEJMoa1009492>

12	The Cardiac Insufficiency Bisoprolol Study II (CIBIS-II): a randomised trial. *Lancet* **353**, 9-13 (1999).

13	Poole-Wilson, P. A. *et al.* Comparison of carvedilol and metoprolol on clinical outcomes in patients with chronic heart failure in the Carvedilol Or Metoprolol European Trial (COMET): randomised controlled trial. *Lancet* **362**, 7-13 (2003). <https://doi.org:10.1016/s0140-6736(03)13800-7>

14	Effect of metoprolol CR/XL in chronic heart failure: Metoprolol CR/XL Randomised Intervention Trial in Congestive Heart Failure (MERIT-HF). *Lancet* **353**, 2001-2007 (1999).

15	McMurray, J. J. *et al.* Angiotensin-neprilysin inhibition versus enalapril in heart failure. *N Engl J Med* **371**, 993-1004 (2014). <https://doi.org:10.1056/NEJMoa1409077>

16	McMurray, J. J. V. *et al.* Dapagliflozin in Patients with Heart Failure and Reduced Ejection Fraction. *N Engl J Med* **381**, 1995-2008 (2019). <https://doi.org:10.1056/NEJMoa1911303>

17	Packer, M. *et al.* Cardiovascular and Renal Outcomes with Empagliflozin in Heart Failure. *N Engl J Med* **383**, 1413-1424 (2020). <https://doi.org:10.1056/NEJMoa2022190>

18	Mebazaa, A. *et al.* Safety, tolerability and efficacy of up-titration of guideline-directed medical therapies for acute heart failure (STRONG-HF): a multinational, open-label, randomised, trial. *Lancet* **400**, 1938-1952 (2022). <https://doi.org:10.1016/s0140-6736(22)02076-1>

19	Swedberg, K. *et al.* Ivabradine and outcomes in chronic heart failure (SHIFT): a randomised placebo-controlled study. *Lancet* **376**, 875-885 (2010). <https://doi.org:10.1016/s0140-6736(10)61198-1>

20	Massie, B. M. *et al.* Toleration of high doses of angiotensin-converting enzyme inhibitors in patients with chronic heart failure: results from the ATLAS trial. The Assessment of Treatment with Lisinopril and Survival. *Arch Intern Med* **161**, 165-171 (2001). <https://doi.org:10.1001/archinte.161.2.165>

21	Pitt, B. *et al.* Spironolactone for heart failure with preserved ejection fraction. *N Engl J Med* **370**, 1383-1392 (2014). <https://doi.org:10.1056/NEJMoa1313731>

22	McDonagh, T. A. *et al.* 2021 ESC Guidelines for the diagnosis and treatment of acute and chronic heart failure: Developed by the Task Force for the diagnosis and treatment of acute and chronic heart failure of the European Society of Cardiology (ESC) With the special contribution of the Heart Failure Association (HFA) of the ESC. *European Heart Journal* **42**, 3599-3726 (2021). <https://doi.org:10.1093/eurheartj/ehab368>

23	Rosano, G. M. C. *et al.* Patient profiling in heart failure for tailoring medical therapy. A consensus document of the Heart Failure Association of the European Society of Cardiology. *Eur J Heart Fail* **23**, 872-881 (2021). <https://doi.org:10.1002/ejhf.2206>

24	Umanath, K., Testani, J. M. & Lewis, J. B. "Dip" in eGFR: Stay the Course With SGLT-2 Inhibition. *Circulation* **146**, 463-465 (2022). <https://doi.org:10.1161/circulationaha.122.060823>

References in the Manuscript

[10] Mebazaa A, Davison B, Chioncel O, Cohen-Solal A, Diaz R, Filippatos G, et al. Safety, tolerability and efficacy of up-titration of guideline-directed medical therapies for acute heart failure (STRONG-HF): a multinational, open-label, randomised, trial. Lancet. 2022;400(10367):1938-52.

[11] Moghaddam N, Hawkins NM, McKelvie R, Poon S, Joncas SX, MacFadyen J, et al. Patient Eligibility for Established and Novel Guideline-Directed Medical Therapies After Acute Heart Failure Hospitalization. JACC Heart Fail. 2023;11(5):596-606.
